# The Fungal Fast Lane: Common Mycorrhizal Networks Extend Bioactive Zones of Allelochemicals in Soils

**DOI:** 10.1371/journal.pone.0027195

**Published:** 2011-11-14

**Authors:** E. Kathryn Barto, Monika Hilker, Frank Müller, Brian K. Mohney, Jeffrey D. Weidenhamer, Matthias C. Rillig

**Affiliations:** 1 Institut für Biologie, Dahlem Center of Plant Sciences, Ökologie der Pflanzen, Freie Universität Berlin, Berlin, Germany; 2 Institut für Biologie, Applied Zoology/Animal Ecology, Freie Universität Berlin, Berlin, Germany; 3 Department of Chemistry, Geology and Physics, Ashland University, Ashland, Ohio, United States of America; Université Joseph Fourier, France

## Abstract

Allelopathy, a phenomenon where compounds produced by one plant limit the growth of surrounding plants, is a controversially discussed factor in plant-plant interactions with great significance for plant community structure. Common mycorrhizal networks (CMNs) form belowground networks that interconnect multiple plant species; yet these networks are typically ignored in studies of allelopathy. We tested the hypothesis that CMNs facilitate transport of allelochemicals from supplier to target plants, thereby affecting allelopathic interactions. We analyzed accumulation of a model allelopathic substance, the herbicide imazamox, and two allelopathic thiophenes released from *Tagetes tenuifolia* roots, by diffusion through soil and CMNs. We also conducted bioassays to determine how the accumulated substances affected plant growth. All compounds accumulated to greater levels in target soils with CMNs as opposed to soils without CMNs. This increased accumulation was associated with reduced growth of target plants in soils with CMNs. Our results show that CMNs support transfer of allelochemicals from supplier to target plants and thus lead to allelochemical accumulation at levels that could not be reached by diffusion through soil alone. We conclude that CMNs expand the bioactive zones of allelochemicals in natural environments, with significant implications for interspecies chemical interactions in plant communities.

## Introduction

Allelopathy, a phenomenon where compounds produced by one plant limit the growth of surrounding plants, is a controversially discussed factor in plant-plant interactions with great potential significance for understanding plant community structure [1], [2], [3], [4]. The potential for allelopathic inhibition of plant growth has been demonstrated repeatedly in the laboratory, but more realistic experiments involving semi-natural soils are often inconclusive [2], [5], [6]. Soil is an extraordinarily complex matrix, and the difficulty of accurately replicating this complexity in the lab may be behind some of the controversial results in allelopathy research [7]. Specific factors relating to soil complexity that are known to influence allelopathic effects include soil moisture [8], organic matter (which adsorbs allelochemicals and reduces availability,[7]), and the presence of microbial communities [9]. These factors often reduce the availability of allelochemicals in soils through sorption, chemical decomposition, and microbial degradation [6]. High rates of microbial degradation [10], [11] are likely especially important in determining allelopathic activities because rates of diffusion of allelochemicals in soils are often low [7], [12], greatly limiting the size of the bioactive zone in which allelochemical levels are high enough to limit growth.

A specific group of soil microbes, the mycorrhizal fungi, are recognized as targets for allelochemicals [13], [14], but no attention has been paid to how these fungi may facilitate transport of allelochemicals through the soil matrix. We propose a mechanism whereby the bioactive zone of allelochemicals could be greatly extended in natural soils due to the occurrence of common mycorrhizal networks (CMNs). We focus on arbuscular mycorrhizal fungi (AMF) and herbaceous plants in this paper, but soils in woody systems also contain CMNs formed by ectomycorrhizal fungi.

More than 80% of vascular plants can form associations with AMF [15], and the relatively low host specificity of these fungi, coupled with frequent formation of anastomoses between intersecting hyphae of the same species, increases the likelihood that one CMN can link multiple plant species in a community [16], [17]. Water and possibly nutrients move between plants *via* the CMN [18], [19], [20], and it is likely that signals inducing plant defenses are also transported [21]. Allelochemical transport *via* CMNs would limit exposure to soil organic matter and reduce sorption and chemical decomposition. Faster transport out of the rhizosphere of the producing plant and passage through the rhizosphere of the target plant, where microbial activity is concentrated [22], would also reduce the time available for microbial degradation. All of these processes could combine to increase the fraction of the allelochemical remaining available at greater distances from the supplier plant. Importantly, CMNs connect neighboring plants, potentially providing direct links from supplier to target plants. Even only allowing for diffusion in the layer of surface water on hyphae, allelochemicals would move more quickly in soil with CMNs than through diffusion in the bulk soil matrix, simply due to the decreased tortuosity of the flow path along the hyphae [23]. Flow rates would be orders of magnitude higher in water flowing on hyphal surfaces [24], or inside hyphae due to cytoplasmic streaming [25].

CMNs are often lacking in manipulative greenhouse experiments because sterile soils or potting media are regularly used in place of natural soils. When natural soils are used experiments are often so short that mycorrhizal networks may not have enough time to fully develop. Simplification of the complex soil matrix is often necessary in order to conduct manipulative experiments, and such approaches have identified many important soil factors contributing to allelochemical activity.

Our objective was to determine whether or not CMNs are a hitherto overlooked, but potentially pivotal factor influencing accumulation of allelochemicals and growth of bioassay plants in natural soils. In greenhouse experiments, we created continuous and interrupted CMNs using two different experimental systems, in order to determine whether or not the bioactive zone of allelochemicals was larger in continuous networks. In both systems we ensured that all plants were mycorrhizal so that any observed differences in growth would not be due to mycorrhizal status, and we verified that nutrient availability did not contribute to any observed differences in growth of bioassay plants. Using first a synthetic herbicide as a model for hydrophilic allelopathic compounds, and then a live plant releasing hydrophobic allelopathic compounds *via* its roots, we found that these compounds accumulated to greater levels in bioassay plants or soils with continuous CMNs than in soils with interrupted fungal networks. In both experiments, these greater levels of allelochemicals were associated with smaller bioassay plants in continuous CMNs than in disrupted fungal networks. CMNs provide a direct route for labile compounds to move from supplier to target plants, suggesting that CMNs may be an overlooked and important factor in the interspecies interactions which may structure some plant communities.

## Materials and Methods

### Experiment 1: A model allelopathic substance

The broad-spectrum, systemic herbicide imazamox (Bolero® 40 g L^−1^ imazamox, BASF Ludwigshafen, Germany, 2-[(*RS*)-4-isopropyl-4-methyl-5-oxo-2-imidazolin-2-yl]-5-methoxymethylnicotinic acid) was used to mimic a phytotoxic allelochemical. Imazamox is water soluble and does not readily adsorb to soil particles, so it should move through the soil matrix, thus being a model compound for comparing diffusion through bulk soil and along CMNs. Furthermore, it is degraded through biotic and abiotic pathways, as are many allelochemicals. Other benefits of using this synthetic phytotoxin as a model allelochemical include its ready availability in large quantities in pure form and extensive information on known bioactive doses. Furthermore, the existence of plant varieties sensitive and resistant to imazamox allowed us to create systems where nurse plants maintaining CMNs were not affected by the phytotoxin.

**Figure 1:**
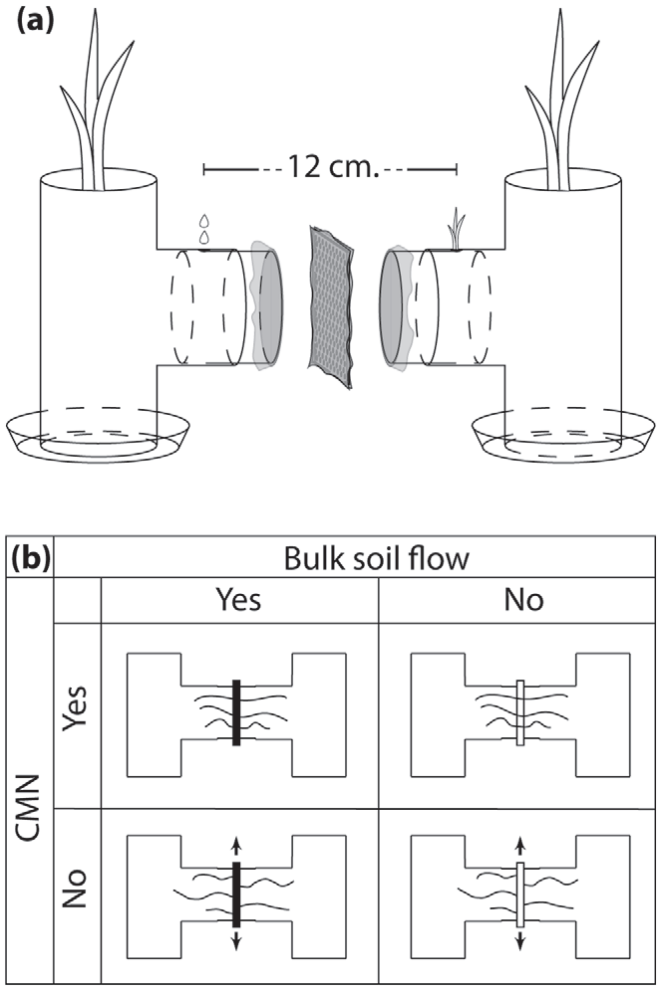
(a) Exploded view of H-pot construction, showing location of large nurse plants, small bioassay plant, the hole where imazamox was introduced to the soil (indicated by droplet symbols), and the four layers of mesh separating pot halves. (b) Experimental design, with solid steel plates indicating that soil was packed into the perforations in the steel plate, while open steel plates were left open creating an air gap. Arrows indicate that plates were moved daily.

Imazamox was applied to one compartment of two-compartment H-bridge pots, and then imazamox levels in the other compartment were assessed both chemically and using a phytometer. We created continuous and interrupted CMNs using custom-designed H-bridge pots constructed of two PVC T-connectors (10.5 cm diameter), PVC tubing (8 cm long, 10.5 cm diameter), 30 µm mesh (Sefar Nitex 03-30/18, Sefar GmbH, Edling, Germany), and perforated steel plates (1.5 mm thick, 12×12 cm) as shown in Fig. 1a. A 1 cm hole was drilled into the horizontal arm of each pot half 6 cm in from the tip to allow introduction of imazamox or a bioassay plant (Fig. 1a). Non-sterile loamy sand collected from an agricultural field at Domäne Dahlem in Berlin, Germany was sieved through a 2-mm sieve, and then packed into pots ensuring that the horizontal arm was also full of soil. The entire H-bridge pot was wrapped several times with tape to prevent movement of the steel plate, and H-bridge pots were not moved during the course of the experiment. Plant roots cannot pass the 30 µm mesh, but fungal hyphae and other soil microbes can, ensuring that the microbial community is consistent throughout the pot.

We investigated imazamox accumulation using a factorial design with diffusion through soil (yes or no) and a CMN (yes or no) as in Fig. 1b. Diffusion through soil was manipulated by filling the steel plate compartment separating pot halves with soil (yes), or leaving the steel plate compartment empty to create an air gap (no). Fungal hyphae easily bridge the air gap [20], allowing for a CMN even with no diffusion through soil. CMNs were destroyed by moving the steel plate daily (approximately 1 cm up and down) to sever any hyphae crossing the mesh (no) or maintained by not moving the steel plate (yes).

Each H-pot was planted with two nurse plants 4 weeks before treatments began in order to establish a CMN. A corn (*Zea mays*) variety resistant to the herbicide imazamox (Clearfield® corn, BASF Ludwigshafen, Germany) was planted in one compartment, and a sensitive variety in the other. The resistant variety was used to ensure that imazamox would not kill the nurse plant in the treated pot compartment. The nurse plant in the untreated half was cut back before bioassays began to ensure that the only plant in that half actively drawing from the CMN was the bioassay plant. In order to minimize water flow across the steel plate we limited saturation of the soil by watering only from the bottom of the pots, and each pot compartment was placed in a separate watering tray.

Imazamox herbicide was applied at a dose equivalent to 200 g active ingredient per hectare by pipetting 5 mL of diluted herbicide into the hole in the horizontal arm of the pot containing the resistant corn plant (Fig. 1a). Two days later the sensitive corn nurse plant was cut at 3 cm above ground level, and then a newly germinated sensitive corn seed was planted in the hole in the horizontal arm of the pot half containing the sensitive corn plant (Fig. 1a). The nurse plant in the imazamox treated half of the pot was not disturbed throughout the experiment. After three weeks seedlings were clipped at ground level, dried, and weighed. The small size of the planting opening (1 cm) made it extremely difficult to remove roots from the soil so we did not attempt to measure belowground biomass. We repeated the experiment using the same established pots because we expected the hyphal network to continue to develop throughout the pot compartments. New sensitive seedlings were planted 2 days after the initial harvest and additional herbicide was applied as before immediately after replanting, and again 1 and 2 weeks after planting, and then seedlings were harvested after 2 weeks. The experiment was repeated a third time, when a set of sensitive seedlings was planted 2 weeks after the second harvest into the same established pots. Herbicide was applied immediately after planting and again 10 days later, and then seedlings were harvested after 2 weeks. At this time soil was collected for analysis of plant available P using the calcium-acetate-lactate method according to the German standard method DIN 3.4.1.30.2a [26], to determine if nutrient levels in pot compartments were affected by treatments. After the final harvest the mesh attached to the steel plates was examined with a dissecting microscope to ensure that mesh had not been damaged during the course of the experiment. Then, squares of mesh were cut from steel plates, placed on microscope slides and stained with several drops of trypan blue (0.05% in 1∶1∶1 glycerol:lactic acid:water). Hyphae on the mesh were examined at 200X to ensure that AMF hyphae were crossing the mesh in the CMN treatments but not in the treatments without a CMN, and that non-AMF hyphae were not abundant.

Above ground material from the third harvest was analyzed for imazamox by HPLC (modified as follows from [27], [28]). HPLC analysis was performed using a Shimadzu LC-20AD with SPD-M20A diode array detector (Shimadzu Deutschland), with a Spherisorb ODS-2 column (4×60 mm, 3 µm) at a flow rate of 0.5 mL min^−1^ (eluent: 0.02 M formic acid:acetonitrile (7:3)). Imazamox identity was confirmed by comparison with retention time (3.2 min) and UV spectra of standards prepared by extracting Bolero® herbicide as for above ground material. A calibration range between 5 and 200 ng imazamox per injected sample was obtained, and all samples fell within this range.

Dry mass of above ground biomass at each harvest, imazamox concentrations in bioassay plants, and plant available soil P concentrations from the third harvest, were analyzed using a factorial ANOVA with diffusion through soil and CMN as factors. Since diffusion through soil was not a significant factor, dry mass from the second and third harvests, and imazamox levels were also analyzed using a one-way ANOVA with CMN as the only factor. Log likelihood tests were conducted to determine which model (two-factor or one-factor) should be used. Data were transformed as necessary to meet assumptions of normality and all analyses were done with R version 2.9.0 [29].

### Experiment 2: Allelopathic substances released from a source plant

**Figure 2:**
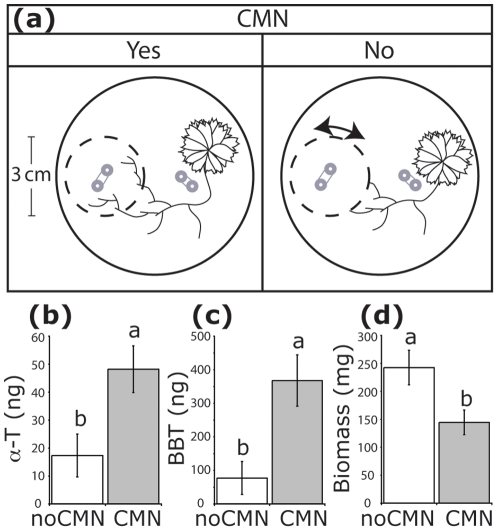
(a) Design of Experiment 2 showing root exclusion compartments (REC) as dashed lines, location of *Tagetes* plant, and *in situ* extraction tubing in gray. The CMN is indicated by black lines, and rotation of the REC prevents CMN formation inside. (b–d) Results from Experiment 2 (means ± SE). Bars with different letters indicate significant differences at α = 0.05. (b) Levels of α-T in *in situ* extraction tubing inside RECs (N = 10). (c) Levels of BBT in *in situ* extraction tubing inside RECs (N = 10). (d) Above ground biomass of bioassay plants in REC soil after initial harvest (N = 11).

A live allelopathic plant, *Tagetes tenuifolia* cultivar “Lemon Gem” (Syringa Samen, Hilzingen, Germany), was chosen for this experiment because it exudes large amounts of the phytotoxic thiophenes 5-(3-buten-1-ynyl)-2,2′-bithienyl (BBT) and α-terthienyl (α-T) from its roots [30]. We focused on the effect of CMNs on allelochemical transport in this experiment, and therefore used a design consisting of root exclusion compartments (REC) that were rotated by approximately 1 cm every other day (no CMN) or left in place (CMN) as in Fig. 2a [31]. RECs were made by covering the sides of 3.5×15 cm filter cylinders (Teichpoint, Mainhausen, Germany) with 30-µm mesh. Seeds were germinated in glass beads before planting in 2-L pots filled with loamy sand from Domäne Dahlem that had been passed through a 4-mm screen prior to use.

Polydimethylsiloxane (PDMS) tubing was used to extract BBT and α-T from soils within and outside of the REC in each pot by *in situ* tube microextraction [32]. Two 1-m lengths of Silastic® tubing (0.30 mm ID×0.64 mm OD, Dow Corning, Midland, MI) were buried in each pot with approximately 2 cm of tubing at each end protruding from the soil surface. Within the REC, tubing was restricted to the inner 1-cm diameter of the soil volume so as to avoid sampling near the mesh. Outside the REC, tubing was restricted to a similar volume of soil, and one *T. tenuifolia* seedling was planted in the center of this coil of tubing.

Pots (10 with a CMN, 10 without) were placed in a climate chamber (18–20°C, 16 hr day length). Eleven weeks after planting, *in situ* tubes were sampled by injecting 1.5 mL of methanol into one end of the tubing at a rate of 1 mL min^−1^ and collecting it as it exited the other end. A 1.5 mL bolus of air was forced through the tubing following the methanol to ensure that all of the solvent was recovered. The methanol was evaporated, and another 1.5 mL of methanol was flushed through the tubing three days later. The methanol was evaporated again, and dry samples were stored at −20°C until analysis. Samples were redissolved in 95% methanol before HPLC analysis [30]. The amounts of thiophenes extracted by this method provide a relative measure of thiophene concentrations in soil, but not a direct measure due to the fact that the soil volume extracted by the tubing is not known.

After extractions were completed, RECs were removed from each pot, all tubing was removed and soil within the REC was thoroughly mixed before being returned to the REC. A further six pots without PDMS tubing, but otherwise equivalent to those used in this experiment, were also harvested, and their soil was used in the bioassay phase as well, bringing the sample size to 13 RECs containing soil that had been conditioned with a CMN and 13 containing soil that not been conditioned with a CMN. A pre-germinated *Lactuca sativa* seedling was planted in each REC, and then harvested 25 days later. Above ground biomass was dried and weighed, and roots were stained with India ink to measure colonization by AMF (modified from [33], [34]). Soil was analyzed for plant available P as in Experiment 1.

Log transformed BBT and α-T levels, biomass and colonization of *L. sativa* seedlings, and log transformed plant available P concentrations were analyzed by one-way ANOVAs with treatment (CMN, no CMN) as the factor. In addition, linear regressions of biomass on thiophene levels were performed to further test if effects on growth were related to changes in thiophene levels. All analyses were done with R version 2.9.0 [29].

## Results

### Experiment 1: A model allelopathic substance

#### The role of diffusion

We compared the importance of diffusion through bulk soil and flow *via* CMNs using a model allelopathic substance, the herbicide imazamox, in custom-designed H-bridge pots where the two pot halves were separated by a perforated steel plate. Diffusion through soil had no significant effects on imazamox concentrations or biomass of target *Zea mays* plants (Table S1); so we also ran more parsimonious models with only CMN as a factor. At the first harvest biomass of target plants was not affected by the presence of either diffusion through soil or CMNs (Table S1), so model simplification was not attempted.

#### The role of CMN

**Figure 3:**
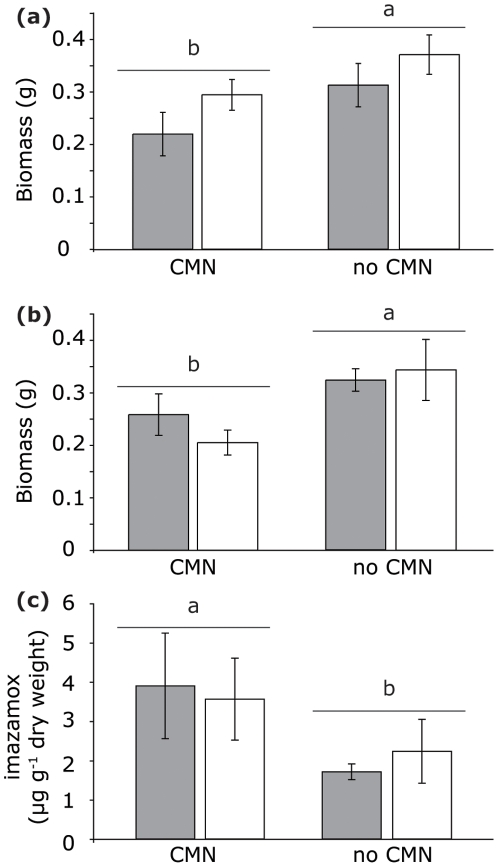
Results from Experiment 1 ± (means SE). Gray bars indicate bulk soil flow, and open bars indicate no bulk soil flow. Bars with different letters indicate significant differences at α = 0.05 with a one-factor (CMN) model. (a) Above ground biomass at harvest 2 (N = 5). (b) Above ground biomass at harvest 3 (N = 5). (c) Imazamox concentrations in leaves at harvest 3 (N = 4–5).

For the second and third harvests, log-likelihood tests supported the simpler models (Table S2). By the second and third harvests, plants were smaller if a CMN was present (harvest 2: *F*_1,18_ = 4.77, *P* = 0.0425; harvest 3: *F*_1,18_ = 7.33, *P* = 0.0144, Fig. 3). Imazamox concentrations in bioassay plants after the third harvest were higher if a CMN was present (*F*_1,16_ = 4.73, *P* = 0.0450, Fig. 3). The significant effects on biomass only in the later harvests suggest that imazamox continued to spread throughout the pot following repeated applications, or that the CMN continued to develop over time facilitating increased accumulation of imazamox. Since imazamox concentrations were low in bioassay plants grown in target compartments lacking CMNs, continued application of imazamox alone cannot explain the increased accumulation of imazamox, and CMNs are clearly contributing to imazamox spread.

#### The role of nutrients

Plant available soil P concentrations were not affected by treatments or their interactions (*P*>0.7, Tables S1, S3), indicating that nutrient levels were likely unaffected by the treatments and that differences in growth of bioassay plants cannot be explained by nutrient differences. We observed intact hyphal networks on mesh screens of CMN treatments, and broken hyphal networks along with clumps of torn hyphae on mesh screens from treatments without a CMN. As with other results, the presence or absence of soil in the steel plate did not appear to affect hyphal structures on the mesh.

### Experiment 2: Allelopathic substances released from a source plant

#### The role of CMN

We conducted a separate experiment using live *Tagetes tenuifolia* plants, which exude allelopathic thiophenes from the roots. Presence of a CMN was manipulated by rotating an REC or leaving it in place. As expected, thiophene abundance outside RECs was not influenced by treatment (overall mean ± SE, α-T: 185±36 ng; *F*_1,13_ = 1.68, *P* = 0.2168; BBT: 1206±155 ng; *F*_1,13_ = 0.01, *P* = 0.913). Inside RECs, abundance of α-T in soils with a CMN was 179% higher than that in soils in RECs without a CMN (*F*_1,18_ = 15.04, *P* = 0.0011), and BBT levels were 378% higher in soils with a CMN than in soils without (Fig. 2b,c; *F*_1,18_ = 16.40, *P* = 0.0008). Above ground biomass of target plants (*Lactuca sativa* seedlings) was smaller when grown in soil from RECs with a CMN, where thiophene levels were highest, than when grown in soil from RECs without a CMN (Fig. 2d; *F*_1,20_ = 14.91, *P* = 0.0010). Four samples were omitted from this analysis because the original seedlings died and had to be replaced, meaning they had a shorter time to grow during the bioassay. However, the difference in biomass remained significant even with these plants included (*F*_1,24_ = 6.67, *P* = 0.0163). Linear regressions revealed a marginally significant negative effect of α-T on biomass of target plants (*F*_1,14_ = 3.19, *P* = 0.0959, *r* ^2^ = 0.19), and a significantly negative effect of BBT on biomass of target plants (*F*_1,14_ = 4.71, *P* = 0.0476, *r* ^2^ = 0.25).

#### The role of nutrients

Plant available soil P concentrations were similar in both treatments (mean ± SE; CMN: 22.9±1.2 mg 100 g^−1^ soil; no CMN: 23.7±1.2 mg 100 g^−1^ soil; *F*_1,22_ = 0.24, *P* = 0.6292), as were AMF colonization rates of bioassay target plants (CMN: 61.4±4.3%; no CMN: 63.3±3.7%; *F*_1,22_ = 0.12, *P* = 0.7335). This indicates that differences in growth of bioassay plants are unlikely to be due to differences in nutrient levels or AMF inoculum potentials inside RECs.

## Discussion

The importance of allelopathy in natural settings is often called into question because of doubts about allelochemicals reaching target plants in high enough doses to be bioactive when they are so often limited by sorption to soil organic matter, chemical decomposition, and microbial degradation [2], [5], [6], [35]. We have shown that organic compounds accumulate to higher levels in soils with intact CMNs than in soils with disrupted networks, and that this increased accumulation translates to reduced growth of target plants. The omission of mycorrhiza from many manipulative experiments may therefore partly explain why allelopathy is often difficult to demonstrate in a soil matrix. We suggest that flow of allelochemicals *via* CMNs may be quite common in the field, and that exchange of these compounds between plants in the field could occur at much higher levels than previously believed.

### Common mycorrhizal networks (CMNs)

We showed that intact CMNs enhanced transport of hydrophilic and lipophilic substances (Exp 1, 2), across a range of distances (Exp 1, 2). Furthermore, this mechanism operated under realistic conditions where allelochemicals were produced by a live plant (Exp 2). Uptake of compounds by target plants was also enhanced by CMNs (Exp 1). Finally, and most importantly, this increased accumulation of noxious compounds was associated with reduced plant growth (Exp 1, 2).

Accumulation of both hydrophilic and lipophilic substances was more pronounced in soils with CMNs than in soils where only diffusion through soil was occurring. Imazamox is hydrophilic (solubility in water: 1777 mg/L, estimated with VCCLAB, http://www.vcclab.org, [36]), and could easily dissolve in the layer of water encircling hyphae. In contrast, both thiophenes are lipophilic, as shown by a high octanol water partition coefficient (log K_o/w_) of 4.98 for α-T [30], and therefore dissolve poorly in water. Small amounts of thiophenes could still be transported in surface water on hyphae, or thiophenes may enter hyphae, either by active uptake or diffusion across cell membranes, followed by active transport around the CMN. Both pathways may be used simultaneously, and their relative importance likely depends on the type of compound.

It is noteworthy that the RECs in Experiment 2 prevented root access to an area with a radius of only 1.5 cm, but that even this short distance was enough to necessitate an intact CMN to ensure delivery of bioactive levels of allelochemicals. In systems where direct root-root contact occurs, such as interactions between the invasive allelopath *Centaurea maculosa* and a native *Festuca*, such contact may also increase delivery of allelochemicals [37]. However, in this system growth of *Festuca* roots not in contact with *C. maculosa* roots also suffered. In plant-plant interactions where root segregation ensures that roots are separated by even one or two centimeters [38], CMNs may be especially important for local chemical interactions and communication between plants. The dependence on intact CMNs for allelochemical transport was also evident at a much greater distance of 12 cm in Experiment 1. It remains to be seen how far compounds spread through natural CMNs, which likely cover even greater distances.

Allelopathy studies are complicated by the difficulty of deciding on a realistic dose for use in experiments. If the chosen dose is too high, then even strong evidence for effects in the greenhouse does not indicate that allelopathy is important in natural settings. In Experiment 2 allelochemicals were produced *in situ* by a live plant, eliminating any concerns about using unrealistic concentrations of externally applied compounds. In addition to enhanced delivery of compounds to the rhizosphere of a target plant, the target plant must also take up the compound in order for allelopathic inhibition to occur. In Experiment 1, we extracted imazamox from target plant tissues, demonstrating that not only does more imazamox reach the rhizosphere of the target plant, but that the target plant also takes it up, a crucial factor in allelopathic inhibition.

Finally, in both experiments the increased accumulation of compounds due to CMNs was associated with significant reductions in plant growth, by 25% and 30% in Experiment 1 (2^nd^ and 3^rd^ harvests) and 40% in Experiment 2. Such reductions would surely affect competitive dynamics in natural settings, with plants sensitive to allelopathic inhibition soon also being outcompeted for light and other nutrients due to their small size. All of these bioassays were short term, 14 or 25 days, and differences in plant growth may increase further with time and continued exposure.

### Other potential contributing factors

Other potential explanations for the observed reductions in plant growth could be a loss of nutrients from soils containing a CMN, a reduction in mycorrhizal inoculum potential, effects of the CMN on bioassay plants, enhanced transport of allelochemicals by other filamentous microbes, or modifications of soil structure by CMNs leading to increased flow of allelochemicals. However, none of these are likely to be important contributors in our system for the reasons detailed below.

Reduced nutrient availability is predicted to lead to reduced plant growth in soils containing a CMN, similarly to increased accumulation of allelochemicals. AMF are especially important for uptake of P [15], and therefore any effects of AMF on nutrient levels should be apparent as changes in plant available soil P. However, we found no differences in soil P availability in either experiment.

Isolation from a CMN is also predicted to reduce mycorrhizal inoculum potential, possibly limiting growth of plants later introduced to such soils due to reduced colonization by the symbiont. Both pot compartments in Experiment 1 contained a plant throughout the experiment in order to maintain the CMN in each pot compartment, even though the pot compartments were not connected by a CMN in all treatments. In Experiment 2, even though the REC did not contain a plant during most of the experiment, bioassay plants were equally colonized regardless of treatment, indicating that mycorrhizal inoculum potential remained high throughout the experiment. This strongly suggests that differences in plant growth were not due to nutrient extraction by intact CMNs, or to reduced inoculum potential of soils separated from CMNs.

Connection to a CMN is known to have variable effects on seedlings, sometimes reducing seedling growth [39], most likely through a disproportionate carbon drain from the seedling to the fungal mycelium [40]. In Experiment 1 both pot halves contained a nurse plant to support the fungi, so at the start of the bioassay target plants could have plugged into a fungal network regardless of treatment, although the network was larger in the CMN treatment because it spanned both pot halves. If growth reductions of bioassay plants had been due to the large carbon costs of supporting the fungal mycelium, we could actually expect greater reductions in the treatment without a CMN where the seedling was the only plant supporting the established fungal mycelium in that pot half. For these reasons we feel that carbon drain to the fungal mycelium is unlikely to have contributed significantly to the growth suppression of seedlings in the CMN treatment in Experiment 1, but we cannot completely rule it out. However, in Experiment 2 the CMN was destroyed before the bioassay so reduced growth of bioassay seedlings could not have been due to carbon drain to the established mycelium. The root exclusion compartments were removed from the pots and the soil inside them was mixed before the bioassay. Target plants were therefore exposed to soil conditioned by a CMN, but they were not supporting an existing mycelium. Colonization of bioassay plants in Experiment 2 was similar with and without a CMN, so we cannot support the idea that the fungi in the CMN treatment were exerting a larger direct carbon cost on those seedlings.

Other filamentous microbes, such as saprophytic fungi and filamentous bacteria, also form branched networks in soils that would have been disrupted during our experiments [22]. These networks could also contribute to the increased accumulation of allelochemicals we observed in soils with CMNs, but are unlikely to be major contributors for several reasons. First, only mycorrhizal fungal hyphae have ever been reported to directly connect roots of different plants. Filamentous bacteria, such as actinobacteria and streptomycetes, acquire energy not from root exudates but from more recalcitrant carbon sources in the soil [22], [41], and are therefore unlikely to form connections between plant roots. Non-mycorrhizal fungi (e.g., saprobes, endophytic or parasitic fungi) can form extensive networks of hyphae in soil [22], [42], but in our system non-AMF hyphae were extremely rare on mesh screens separating pot compartments suggesting that, independent of overall abundance, their potential contribution to bridging between compartments was negligibly low.

Modifications of the soil environment by fungal hyphae could also indirectly contribute to these effects by altering soil structure [43] to enhance hydraulic conductivity [44], and by reducing microbial degradation of compounds. However, bacterial biofilms are common on hyphae [45], demonstrating that hyphae are not broadly antimicrobial habitats. Any indirect alteration of the soil by hyphae would have occurred in both pot compartments in Exp 1, where the pot was completely colonized by mycorrhizal fungi, even in treatments where pot compartments did not share the same CMN. This strongly suggests that the connectivity of the hyphal network is of primary importance, and not simply the presence of fungal hyphae.

### Conclusions

We are opening the door to a field of inquiry crucial for understanding allelopathy in natural systems. Clearly, there is room for much future research on the importance of CMNs for allelopathic interactions between plants. Immediate questions relate to localization of allelochemicals to hyphal surfaces or interiors, determination of flow rates, the importance of any direct inhibition of hyphal growth by allelochemicals, and how non-mycorrhizal supplier and target plants are affected by CMNs. There are unlikely to be simple answers to these questions, as many responses will vary with allelochemical. However, many should be predictable based on chemical characteristics (e.g., hydrophilicity, size) and much focused research in the near future could address many of these important questions.

Mediation of plant-plant communication in the field will depend on the extent to which CMNs link conspecific or heterospecific plants. Links between conspecifics are suggested by the growing body of literature demonstrating preferential plant fungus pairings [46], [47]. However, links between heterospecifics have also been regularly demonstrated [16]. Of particular interest for the allelopathic interactions described here is the fact that an invasive allelopathic plant (*Centaurea maculosa*) can override fungal preferences in surrounding plants and ensure that they share its fungal community, and therefore network [48]. It remains to be seen how widespread this phenomenon of controlling the CMN of neighboring plants is among allelopathic plants.

We have presented strong evidence that intact CMNs can expand the bioactive zone of allelochemicals in soil by facilitating their transport, thereby ‘rescuing’ the function of allelochemicals released into hot spots of microbial activity in soils. Interplant interactions below ground include not only allelopathic interactions mediated by allelochemicals, but also interactions involving plant hormones and signaling compounds that move through the soil matrix. Passage *via* CMNs may facilitate these functions by expanding the effective bioactive zone for all of these compounds, and it is therefore likely that the importance of CMNs for interplant interactions and communication in natural settings has not yet been fully recognized.

## Supporting Information

Table S1 **Statistical results of two-factor model analysis of Experiment 1.**
(DOC)

Table S2 **Results of log-likelihood tests for model simplification in Experiment 1.**
(DOC)

Table S3 **Experiment 1 soil P concentrations (mean ± SE) in bioassay soils at the end of the experiment.**
(DOC)
