# Supplementary material for: The Fungal Fast Lane: Common Mycorrhizal Networks Extend Bioactive Zones of Allelochemicals in Soils
Source: PLoS One. 2011 Nov 14;6(11):e27195. doi: 10.1371/journal.pone.0027195 (PMC3215695; doi:10.1371/journal.pone.0027195)
Supplement: Table S1 — Statistical results of two-factor model analysis of Experiment 1. (DOC) [file pone.0027195.s001.doc]

**Table S1** Statistical results of two-factor model analysis of Experiment 1 shoot biomass (three harvests), imazamox concentrations in bioassay plant leaves (final harvest), and soil P at the end of the experiment (N = 2 - 5).

|  |  | Biomass  (harvest 1) | | Biomass  (harvest 2) | | Biomass  (harvest 3) | | Imazamox  (harvest 3) | | Soil P  (harvest 3) | |
| --- | --- | --- | --- | --- | --- | --- | --- | --- | --- | --- | --- |
|  | df | *F* | *P* | *F* | *P* | *F* | *P* | *F* | *P* | *F* | *P* |
| Bulk soil flow | 1 | 0.71 | 0.4121 | 3.10 | 0.0972 | 0.19 | 0.6712 | 0.01 | 0.9228 | 0.09 | 0.7711 |
| CMN | 1 | 1.00 | 0.3330 | 5.08 | 0.0387 | 6.96 | 0.0179 | 4.14 | 0.0611 | 0.01 | 0.9390 |
| Interaction | 1 | 1.12 | 0.3061 | 0.05 | 0.8278 | 0.90 | 0.3577 | 0.0002 | 0.9897 | 0.15 | 0.7099 |
|  |  | Error df = 15 | | Error df = 16 | | Error df = 16 | | Error df = 14 | | Error df = 9 | |
