# Supplementary material for: The Fungal Fast Lane: Common Mycorrhizal Networks Extend Bioactive Zones of Allelochemicals in Soils
Source: PLoS One. 2011 Nov 14;6(11):e27195. doi: 10.1371/journal.pone.0027195 (PMC3215695; doi:10.1371/journal.pone.0027195)
Supplement: Table S2 — Results of log-likelihood tests for model simplification in Experiment 1. (DOC) [file pone.0027195.s002.doc]

**Table S2** Results of log-likelihood tests for model simplification in Experiment 1.

|  | Biomass  (harvest 2) | Biomass  (harvest 3) | Imazamox  (harvest 3) |
| --- | --- | --- | --- |
| Log-likelihood of two-factor model | 23.309 | 22.846 | 9.712 |
| Log-likelihood of one-factor model | 21.510 | 22.191 | 9.706 |
| Log-likelihood *P* ^a^ | 0.237 | 0.592 | 0.995 |

^a^ A *P*-value greater than 0.05 indicates support for the simpler model.
