# Supplementary material for: The Fungal Fast Lane: Common Mycorrhizal Networks Extend Bioactive Zones of Allelochemicals in Soils
Source: PLoS One. 2011 Nov 14;6(11):e27195. doi: 10.1371/journal.pone.0027195 (PMC3215695; doi:10.1371/journal.pone.0027195)
Supplement: Table S3 — Experiment 1 soil P concentrations (mean ± SE) in bioassay soils at the end of the experiment. (DOC) [file pone.0027195.s003.doc]

**Table S3** Experiment 1 soil P concentrations (mean ± SE) in bioassay soils at the end of the experiment (N = 2 – 4).

| Bulk soil flow | CMN | Soil P (mg 100 g^-1^ soil) |
| --- | --- | --- |
| Yes | Yes | 9.7 ± 1.0 |
| Yes | No | 8.6 ± 1.3 |
| No | Yes | 9.8 ± 1.9 |
| No | No | 11.2 ± 3.3 |
